# Detection of differential DNA methylation in date palm (*Phoenix dactylifera* L.) vitroplants using whole genome bisulfite sequencing analysis

**DOI:** 10.1186/s12870-026-09190-6

**Published:** 2026-06-10

**Authors:** Imene Mattat, Ayeda A. Ahmed, Shameem Younuskunju, Krunal Pawar, Yasmin A. Mohamoud, Ameena A. Almalki, Joel A. Malek, Walid Hamada

**Affiliations:** 1 Biotechnology Section, Agriculture Research Department, Ministry of Municipality, Doha, Qatar; 2 Genomics Core, Weill Cornell Medicine–Qatar, Doha, Qatar; 3 Sedeer Medical, Diagnostic and Research, Doha, Qatar; 4 Institut National Agronomique de Tunisie (INAT), Tunis, Tunisia

**Keywords:** DNA methylation, Tissue culture, Date palm, Whole Genome Bisulfite Sequencing (WGBS)

## Abstract

Date palm (*Phoenix dactylifera* L.) is an essential fruit crop in arid and semi-arid regions, where tissue culture is widely used for large-scale clonal propagation. However, this technique often generates somaclonal variants that deviate from original cultivar traits, largely due to epigenetic modifications such as DNA methylation. Here, whole genome bisulfite sequencing (WGBS) was applied to two widely cultivated Qatari cultivars, Khalas and Kheneizi, to compare cytosine methylation profiles between tissue culture-derived plants and offshoot-derived controls. Thousands of differentially methylated regions (DMRs) were identified. Khalas exhibited a predominance of hypomethylated regions, whereas Kheneizi showed slightly more hypermethylated regions. Across both cultivars, CG sites tended to lose methylation, while CHG sites more frequently gained methylation in tissue culture-derived plants. Genes overlapping with DMRs (DMGs) displayed cultivar-specific enrichment patterns. In Khalas, hypermethylated DMGs were mainly linked to nuclear organization, chromatin remodeling, and stress signaling, while hypomethylated genes were associated with defense responses and intercellular communication. In Kheneizi, hypermethylated DMGs were enriched in RNA metabolism, translation, and hormone signaling, whereas hypomethylated DMGs were associated with metabolism, phosphorylation-mediated regulation, and stress responses. KEGG enrichment further revealed shared involvement of RNA degradation, mRNA surveillance, aminoacyl-tRNA biosynthesis, and MAPK signaling pathways. These results demonstrate that tissue culture induces DNA methylation changes in date palm, potentially affecting development and contributing to off-type phenotypes. Understanding these epigenetic alterations provides valuable insights for improving clonal fidelity and optimizing tissue culture propagation in perennial crops.

**Supplementary Information:**

The online version contains supplementary material available at 10.1186/s12870-026-09190-6.

## Introduction

The date palm (*Phoenix dactylifera* L.) occupies a significant place in human history as one of the oldest cultivated fruit trees. It serves as a crucial source of sustenance and economic prosperity, particularly in arid and semi-arid regions. These regions have seen the date palm become an integral component of their traditions, economies, and ecosystems. This is due to its remarkable ability to thrive even in regions with limited water resources [1].

Its potential to contribute significantly to food security in arid areas has been recognized. To unlock this potential, researchers and agriculturalists are actively exploring innovative cultivation practices. These practices encompass the adoption of modern irrigation technologies [2], advanced breeding programs, and refined crop management strategies [3], all aimed at optimizing date palm yields and enhancing the quality of the product.

Traditionally, date palm has been propagated through offshoots. However, the advancement of propagation techniques through tissue culture has led to a significant expansion of date palm plantations [4].

The development of tissue culture in the 1970s and its widespread application for date palm production up to the present day has greatly contributed to increasing production levels meeting high demand through large-scale clonal propagation [5].

Although both offshoot propagation and in vitro tissue culture involve developmental reprogramming, they differ substantially in their regulatory and physiological conditions. Offshoot-derived plants develop in planta under endogenous hormonal regulation and natural environmental conditions [6], whereas in vitro propagation involves prolonged dedifferentiation and re-differentiation phases under artificial culture conditions, often requiring high and sustained levels of exogenous plant growth regulators, particularly auxins and cytokinins [7].

Tissue culture propagation has the ability to produce a substantial number of true-to-type plants identical to their progenitors. Unfortunately, in vitro date palm propagation methods often lead to the production of off-types that deviate from the characteristics of the original cultivar. These are somaclonal variations generated by mutations and/or epigenetic changes happening during the process of multiplication [8].

The use of artificial hormonal environments induces the resetting of the cells’ genetic and epigenetic patterns and often leads to epigenetic events and the formation of somaclone variants [9]. In fact, the use of higher concentrations of auxins such as 2,4-D has been shown to be associated with this phenomenon [10].

Among epigenetic mechanisms, DNA methylation plays a pivotal role as an epigenetic regulator in plant tissue culture, influencing tissue specificity and cellular responses to in vitro conditions. It involves the addition of a methyl group to cytosine bases in CpG, CHG, or CHH (where H = A, C, or T) contexts [11]. Cytosine methylation levels are dynamically regulated by DNA methylation and demethylation reactions [12].

Gain and loss of DNA methylation can occur in response to tissue culture stress induced by culture conditions such as auxin or cytokinin treatments, nutrient media composition, and mechanical handling of cells [13], which influence somatic embryogenesis and organogenesis [14]. Increased methylation often leads to gene silencing, particularly in transposable elements and promoter regions, whereas reduced methylation can activate gene expression, influencing traits such as growth and stress responses [15].

These epigenetic changes can stabilize or disrupt developmental programs, thereby contributing to somaclonal variation, a common phenomenon in plant tissue culture. Moreover, tissue-specific methylation patterns are frequently reprogrammed during dedifferentiation and regeneration, affecting the expression of genes involved in stress responses, growth, and differentiation [16].

In oil palm (*Elaeis guineensis* Jacq.), a correlation has been demonstrated between DNA hypomethylation and the mantled somaclonal variation, a tissue culture-related disorder in which somatic embryo-derived palms display floral developmental abnormalities, notably the feminization of male reproductive organs [17]. This association was further confirmed by Ong-Abdullah et al. [18], who identified significant hypomethylation of the Karma transposon in the mantled oil palm phenotype.

Understanding DNA methylation changes in tissue culture systems is critical for improving clonal fidelity and enhancing the efficiency of plant propagation. In this study, we investigated genome-wide DNA methylation patterns using bisulfite sequencing to identify propagation-associated DNA methylation differences between phenotypically normal tissue culture-derived and offshoot-derived plants, independent of visible abnormalities.

Whole-genome bisulfite sequencing (WGBS) enables single-base resolution detection of DNA methylation by identifying methylated cytosines across the entire genome, including within individual chromosomes, and is currently considered the highest-resolution approach for genome-wide methylome analysis [19].

WGBS relies on bisulfite treatment of genomic DNA, during which unmethylated cytosines are converted to uracil and subsequently read as thymines after sequencing, whereas methylated cytosines remain protected and are retained as cytosines [20].

Using this approach, differentially methylated regions (DMRs) were identified across the date palm genome, and genes associated with altered methylation were subsequently characterized. This strategy enabled the detection and investigation of propagation-associated DNA methylation patterns in tissue culture-derived plants relative to offshoot-derived controls and facilitated the identification of candidate genomic regions potentially affected by in vitro propagation.

## Materials and methods

### Plant materials

Two widely cultivated date palm (*Phoenix dactylifera* L.) cultivars in Qatar, Kheneizi and Khalas, were selected for this study due to their high preference among both growers and consumers, attributed to their superior yield and fruit quality. Fresh leaf samples were collected from adult, reproductive-stage date palm plants derived via in vitro somatic embryogenesis and from conventionally propagated offshoots at the Agricultural Research Department experimental farm, Rawdat Al-Faras Research Station (Ministry of Municipality, Doha, Qatar).

At the time of sampling, phenotypic assessment was conducted, and samples were collected exclusively from “normal” trees exhibiting no visible morphological or developmental abnormalities, including abnormal leaf morphology, growth retardation or dwarfism, chlorosis or necrosis, deformities, atypical tillering, or other developmental defects, and showing growth patterns consistent with their respective cultivars at the sampled stage. All samples were collected under identical cultivation conditions. Sampling was performed randomly within uniform date palm plantations exhibiting similar phenotypic traits. All samples were immediately frozen in liquid nitrogen and stored at − 80 °C until further processing (Table 1).

**Table 1:** Date palm samples

Tube number	Code	Cultivar	Type
4	KZ4	Kheneizi	TC (normal)
5	KZ5	Kheneizi	TC (normal)
6	KZ6	Kheneizi	TC (normal)
7	KZ7	Kheneizi	Offshoot (normal)
8	KZ8	Kheneizi	Offshoot (normal)
9	KZ9	Kheneizi	Offshoot (normal)
52	KH10	Khalas	TC (normal)
53	KH11	Khalas	TC (normal)
54	KH12	Khalas	TC (normal)
25	KH19	Khalas	Offshoot (normal)
26	KH20	Khalas	Offshoot (normal)
27	KH21	Khalas	Offshoot (normal)

All samples were collected at the Agricultural Research Department (ARD) experimental farm, Rawdat Al-Faras Research Station (Ministry of Municipality, Doha, Qatar). TC (normal) = vitroplants regenerated via somatic embryogenesis; Offshoot (normal) = conventionally propagated offshoots

Collections were conducted under institutional authorization of the Agriculture Research Department at its own research station. No wild plants or protected species were involved, and no additional collection permits were required. All experimental work on cultivated plants complied with institutional and national guidelines and was conducted in accordance with applicable local legislation.

### Library construction and whole genome bisulfite sequencing

#### Genomic DNA isolation

Genomic DNA was extracted according to the DNeasy Plant Mini kit (Qiagen Venlo, The Netherlands) according to the manufacturer’s instructions. The extracted DNA was accurately and quickly quantified using The Invitrogen Qubit 2.0 Fluorometer (Life Technologies, CA, United States).

#### Bisulfite conversion and library preparation

First, 100 ng of genomic DNA was fragmented using sonication to 300 bp with a Covaris S220 (Covaris, Woburn, MA, United States).

The fragmented DNA was then bisulfite-converted using the EZ DNA Methylation-Gold Kit (Zymo Research, Irvine, CA, United States) following the manufacturer’s instructions. The remainder of the library preparation followed the manufacturer’s protocol as described in xGen™ Methyl-Seq Library Prep for Illumina. Briefly, the bisulfite-converted DNA was A-tailed and end-repaired to generate blunt ends, then Illumina adaptors were ligated; finally, the adaptor-ligated DNA fragments were indexed with xGen™ UDI Primers using 5 cycles of PCR enrichment. The libraries were purified using 0.85x SPRISelect beads. The quality and quantity of the final NGS libraries were assessed using Bioanalyzer High Sensitivity chips and KAPA Library Quantification Kit for Illumina.

#### Sequencing

Finally, the libraries were pooled in equimolar ratios and sequenced on Illumina NovaSeq 6000 S4 v1.5 in a paired-end 150 bp run.

#### Reads mapping to the reference genome

The millions of reads generated by the sequencer were aligned to the date palm PDK50 reference genome (NCBI BioProject ID: PRJNA40349) using Bismark (v0.24.1) pipeline, the bisulfite read aligner [21].

Each tissue culture-derived and offshoot-derived sample was processed independently throughout the Bismark workflow including genome preparation, alignment, deduplication, and methylation extraction.

Prior to alignment, raw reads were trimmed and quality-filtered with fastp (v0.19.4) [22], removing 5 and 10 bases from the start and end of R1, and 10 and 5 bases from the start and end of R2, respectively. Trimmed reads were aligned to the bisulfite-converted reference genome with Bismark (--score_min L,0,-0.4), followed by PCR duplicate removal using deduplicate_bismark. Methylation calls were generated with bismark_methylation_extractor (--cytosine_report, --comprehensive, --CX), and the resulting CX reports were used as input for DMR analysis.

### DNA methylation analysis and detection of differentially methylated regions and their related genes

DNA methylation analysis and identification of differentially methylated regions (DMRs) were carried out using the Bismark pipeline (v0.24.1) [21] and the DMRcaller R package [23].

Global DNA methylation levels for CG, CHG, and CHH contexts were calculated for each sample based on cytosine methylation reports generated by the Bismark pipeline. For each context, the number of methylated cytosines was divided by the total number of cytosines analyzed across the genome to obtain genome-wide methylation percentages. Mean methylation levels were then calculated for tissue culture-derived and offshoot-derived samples for each cultivar.

DMRs were identified using the ‘bins’ method implemented in the DMRcaller R package, which evaluates methylation at individual cytosine positions and aggregates methylation differences across defined genomic windows. Pairwise comparisons were performed between offshoot-derived (normal) and tissue culture-derived (TC; normal) samples for both Khalas and Kheneizi cultivars.

DMRs were first identified separately for each cultivar (Khalas and Kheneizi). For the combined Khalas-Kheneizi analysis, samples were grouped by propagation method, such that tissue culture-derived samples from both cultivars were combined into a single group and compared exclusively against a combined group of offshoot-derived samples.

Propagation method was tested as the primary biological factor, while cultivar was not treated as an independent factor or interaction term; separate and combined analyses were used to evaluate the consistency of propagation-associated DNA methylation changes across distinct genetic backgrounds. DMR detection was based on aggregated methylation profiles at the group level; intragroup variability among individual samples was therefore not explicitly quantified.

DMR identification was performed using stringent filtering criteria, including a minimum intergroup methylation difference of ≥ 50%, to minimize technical noise and enhance the robustness of the detected methylation differences.

Separate analyses were conducted for the CG and CHG methylation contexts using the parameters summarized in Table 2. Methylation levels were calculated as the proportion of methylated cytosines over total cytosines covered within each bin, and statistical significance was assessed using Fisher’s exact test; DMRs with adjusted p (FDR) ≤ 0.01 were considered significant.

**Table 2:** Parameters used for DMR identification

Method	bins
window size (bp)	100
PvalueThreshold	0.01
minCytosinesCount	4
minProportionDifference	0.5
minGap	200
minSize	50
minReadsPerCytosine	4

DMR boundaries were extended up to 1 kb upstream of each gene to capture potential regulatory elements.

Following the identification of DMRs in each methylation context, the differential methylation ratio attributable to the tissue culture (TC) process was calculated by subtracting the methylation proportion in offshoot samples from the methylation proportion in TC vitroplants. DMRs were further classified based on the direction of methylation change: hypermethylated DMRs exhibit increased methylation in TC vitroplants compared to offshoots, while hypomethylated DMRs show decreased methylation. The overall methylation change during the TC process was quantified using the formula:

$$\begin{aligned}&((\mathrm{Number}\:\mathrm{of}\:\mathrm{hypermethylated}\:\mathrm{DMRs}\\&-\mathrm{Number}\:\mathrm{of}\:\mathrm{hypomethylated}\:\mathrm{DMRs})\\&/\mathrm{Total}\:\mathrm{number}\:\mathrm{of}\:\mathrm{significant}\:\mathrm{DMRs})\times100\end{aligned}$$

Chromosomal distribution of DMRs was assessed by assigning each DMR to its corresponding linkage group (LG) based on genomic coordinates. For each chromosome, the proportion of hypermethylated and hypomethylated DMRs was calculated separately for each cultivar and methylation context.

To examine the distribution of differential methylation across genomic features, DMRs were classified according to their overlap with annotated gene bodies or transposable elements (TEs) based on the PDK50 genome annotation.

Differentially methylated region-associated genes (DMGs) were defined as those whose gene body or upstream regions overlapped with one or more DMRs.

### Functional enrichment analysis of DMGs

To functionally annotate genes associated with hyper- and hypomethylated regions in both cultivars, differentially methylated regions (DMRs) in BED format were first processed by standardizing chromosome identifiers to match the *Phoenix dactylifera* PDK50 reference genome (assembly GCA_000181215.3). Gene-associated DNA sequences were retrieved using bedtools getfasta. Homology searches were performed with DIAMOND blastx against a locally constructed protein database from the PDK50 reference proteome, using an E-value cutoff of 1 × 10⁻⁵ and retaining only the top-scoring hit per query. The resulting protein identifiers were harmonized, and full-length protein sequences were retrieved with SeqKit [24, 25]. Functional annotation and enrichment analysis were conducted in OmicsBox, where EggNOG-mapper was applied to assign Gene Ontology (GO) terms and KEGG pathway annotations. GO and KEGG enrichment were assessed using Fisher’s exact test; significance was set at *p* < 0.05, employing the complete PDK50 proteome as the reference background [26–29].

## Results

### DNA methylation data

Whole-genome bisulfite sequencing (WGBS) was performed on 12 samples, including six normal tissue culture–derived vitroplants and six normal offshoots, and methylation variations were predominantly detected at CG and CHG sites, while CHH-context DMRs were observed exclusively in the Kheneizi cultivar. Sequencing depth per sample ranged from approximately 25× to 35×, with the lowest coverage observed in sample KZ6 (25.47×) and the highest in KH10 (34.85×) (Table 1). Bisulfite conversion rates were consistently high across all samples, ranging from 92.0% to 93.05%, indicating efficient conversion of unmethylated cytosines. Mapping efficiency ranged from 63.7% to 70.6% across all samples, with consistent values observed among biological replicates, indicating reliable alignment of bisulfite-treated reads to the reference genome (Table 3). These quality metrics ensured reliable methylation calling, consistent performance across biological replicates, and robust identification of differentially methylated regions (DMRs) between the two groups.

**Table 3:** Sequencing depth and bisulfite conversion rates

Sample ID	KZ4	KZ5	KZ6	KZ7	KZ8	KZ9	KH10	KH11	KH12	KH19	KH20	KH21
Depth	34.22	31.19	25.47	29.26	25.79	31.87	34.84	26.72	29.66	28.49	34.22	28.99
Bisulfite conversion (%)	92.73	92.63	92.87	92.51	92.69	93.05	92.00	92.25	92.47	92.00	92.59	92.60
Mapping Efficency (%)	63.7	67.3	67.4	64.4	64.2	66.6	65.7	69.5	69.6	69.2	70.6	70.4

These quality metrics ensured reliable methylation calling and robust identification of differentially methylated regions (DMRs) between the two groups.

**Fig. 1:**
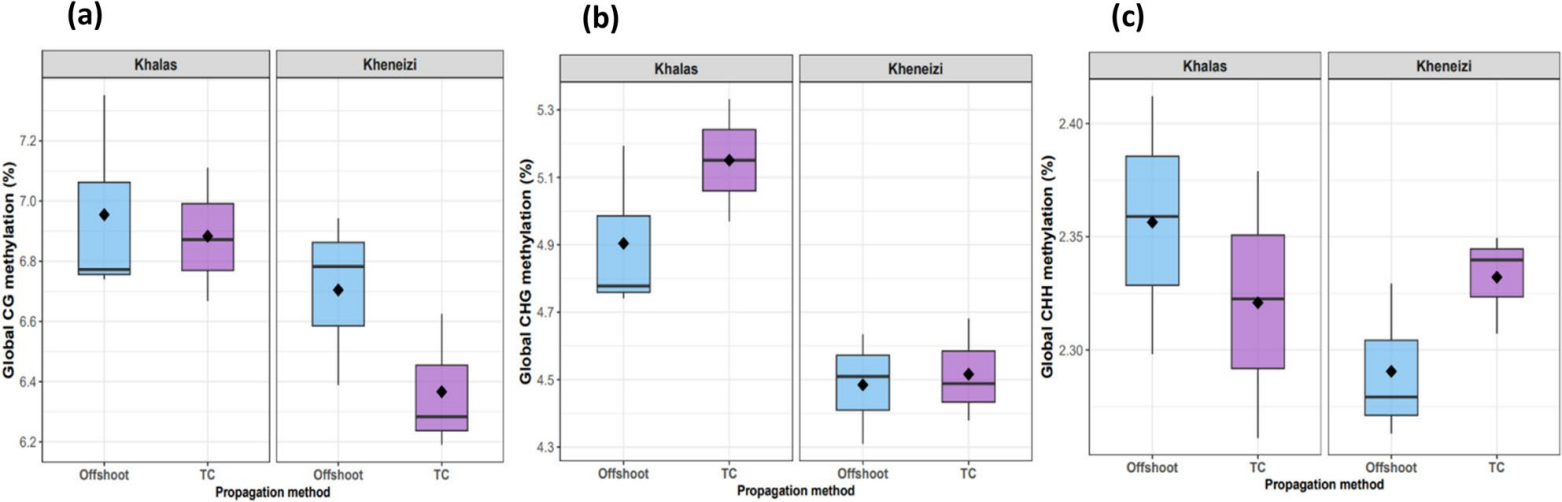
Global DNA methylation levels across cytosine contexts in Khalas and Kheneizi cultivars.Genome-wide methylation percentages in the CG (**a**), CHG (**b**), and CHH (**c**) contexts are shown for offshoot-derived and tissue culture-derived plants. Boxes represent the interquartile range, center lines indicate the median, whiskers extend to 1.5× the interquartile range, and black diamonds denote mean methylation levels

Genome-wide DNA methylation levels showed minor differences between tissue culture-derived and offshoot-derived plants across CG, CHG, and CHH contexts in both cultivars (Fig. 1). In the Khalas cultivar, tissue culture-derived plants exhibited a slightly lower mean CG methylation level compared with offshoot-derived controls (6.88% vs. 6.95%), whereas CHG methylation levels were marginally higher in tissue culture-derived plants (5.15% vs. 4.90%). CHH methylation represented a minor fraction of total methylation and was slightly reduced in tissue culture-derived plants relative to offshoot-derived plants (2.32% vs. 2.36%).

A similar pattern was observed in the Kheneizi cultivar with respect to CG and CHG contexts, where tissue culture-derived plants displayed reduced CG methylation compared with offshoot-derived plants (6.37% vs. 6.70%) and slightly elevated CHG methylation levels (4.52% vs. 4.48%).

In contrast to Khalas, CHH methylation represented a minor proportion of total methylation but was marginally higher in tissue culture-derived plants than in offshoot-derived plants (2.33% vs. 2.29%).

### Identification of the differentially methylated regions (DMRs)

The differentially methylated regions (DMRs), showing differences in methylation levels between tissue-culture vitroplants and offshoot controls for each cultivar and for the combined Khalas–Kheneizi cultivars, were identified. Analyses were performed separately for the CG, CHG and CHH contexts, and DMRs were considered significant at adjusted *p* (FDR) ≤ 0.01.

A total of 3,880, 10,493, and 450 DMRs were significantly altered (adjusted *p* ≤ 0.01) in the Khalas, Kheneizi, and combined Khalas-Kheneizi groups, respectively, in response to tissue culture (Table S9).

Statistical analysis showed that CG-context DMRs were predominant across all groups, accounting for 53.58%, 75.83%, and 51.55% in Khalas, Kheneizi, and the combined dataset, respectively, compared to CHG context DMRs, which represented 46.42%, 24.03%, and 48.45% (Table 4).

**Table 4:** Counts and percentages of significant DMRs (adjusted *p* ≤ 0.01) in CG and CHG contexts; overall methylation changes (%) computed on CG + CHG combined

		mCG		mCHG		mCHH		Overall methylation change (%)
		No.	%	No.	%	No.	%	
KHALAS	Hypermethylated DMRs	150	7.21	1455	80.79	-	-	-17.26
	Hypomethylated DMRs	1929	92.79	346	19.21	-	-	
	Methylation changes at DMRs (%)	-	-85.55	-	61.57	-	-	
KHENEIZI	Hypermethylated DMRs	3378	42.45	2020	80.1	5	35.71	2.98
	Hypomethylated DMRs	4579	57.54	502	19.9	9	64.29	
	Methylation changes at DMRs (%)	-	-15.09	-	60.2	-	-28.58	
KHALAS- KHENEIZI	Hypermethylated DMRs	43	18.53	192	88.07	-	-	4.44
	Hypomethylated DMRs	189	81.47	26	11.93	-	-	
	Methylation changes at DMRs (%)	-	-62.93	-	76.14	-	-	

Based on the significant differences in methylation ratio between tissue culture-derived normal plants and offshoot-derived controls, approximately 41.36% of DMRs in the Khalas cultivar were hypermethylated, while 58.63% were hypomethylated. In contrast, the Kheneizi cultivar showed that 51.49% of DMRs being hypermethylated and 48.51% hypomethylated. A similar trend was observed in the combined dataset, with 52.22% hypermethylated and 47.78% hypomethylated DMRs (Table 4).

Interestingly, in all three datasets, hypermethylated DMRs were more frequent than hypomethylated ones in the CHG context. However, in the CG context, hypomethylated DMRs were more abundant than their hypermethylated counterparts. These findings suggest that DNA methylation in CG regions tends to decrease, whereas methylation in CHG regions tends to increase, in plants regenerated through tissue culture (Table 4).

Notably, CHH-context DMRs were detected exclusively in the Kheneizi cultivar and represented a minor fraction of total DMRs, with a predominance of hypomethylation.

**Fig. 2:**
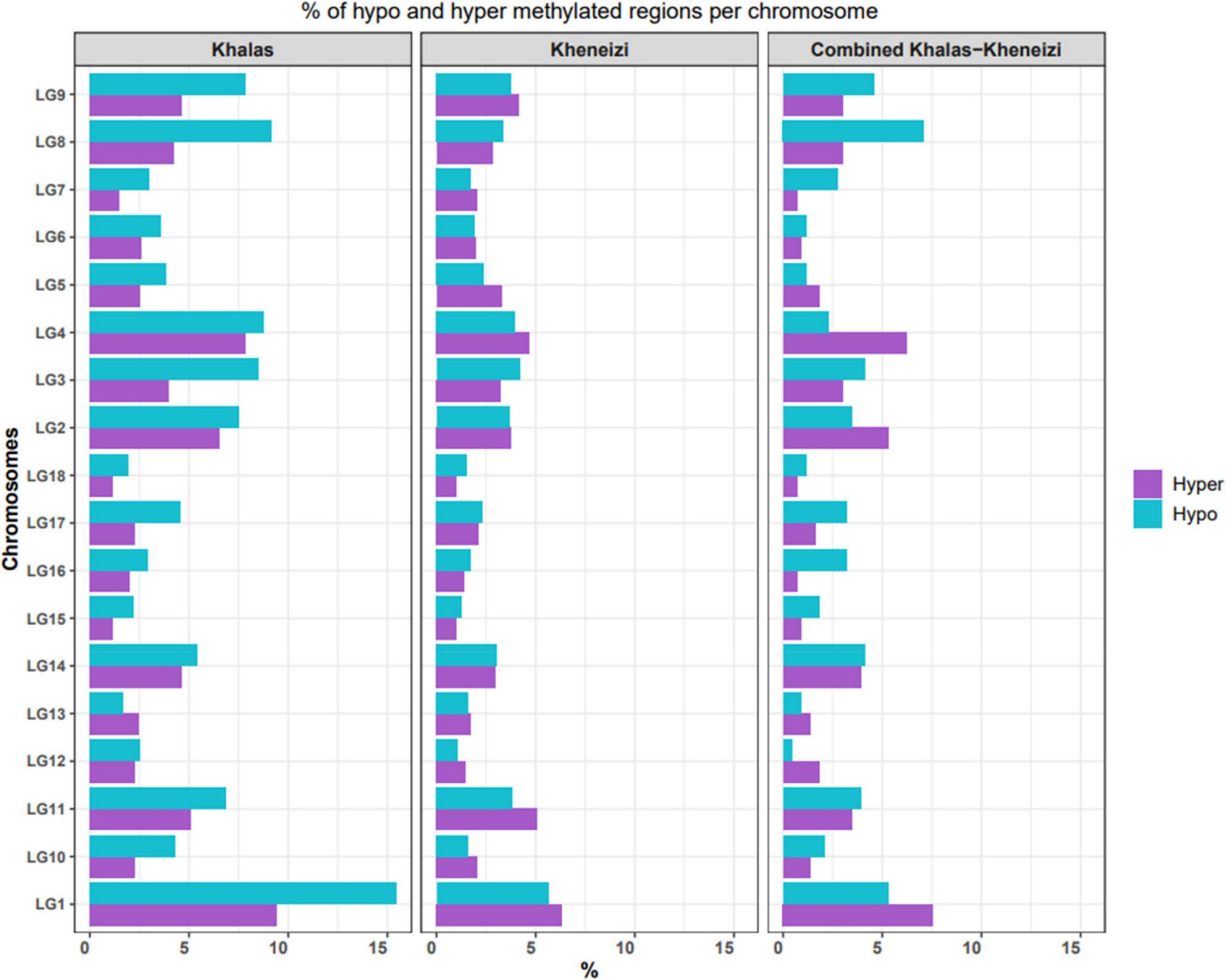
Chromosomal distribution of hypermethylated and hypomethylated DMRs in Khalas, Kheneizi, and the combined dataset. The proportion (%) of hypermethylated and hypomethylated DMRs was calculated for each linkage group (LG1–LG18) in the Khalas cultivar, the Kheneizi cultivar, and the combined Khalas–Kheneizi dataset. DMRs were classified as hypermethylated or hypomethylated based on methylation differences between tissue culture-derived and offshoot-derived plants. Percentages represent the relative contribution of DMRs per chromosome within each dataset. Colors indicate hypermethylated and hypomethylated DMRs, as shown in the legend

Differentially methylated regions (DMRs) were distributed across all 18 linkage groups in both cultivars, with distinct chromosomal patterns observed for hypermethylated and hypomethylated regions (Fig. 2). DMRs were not uniformly distributed along the chromosomes, and the relative contribution of individual linkage groups varied between cultivars.

In the Khalas cultivar, hypomethylated DMRs predominated across most chromosomes, with particularly high proportions observed on LG1, LG4, LG3, LG8, and LG11. Hypermethylated DMRs were detected on all chromosomes but generally occurred at lower frequencies compared with hypomethylated regions.

In contrast, the Kheneizi cultivar showed an opposite trend, with hypermethylated DMRs being more frequent than hypomethylated DMRs across the majority of chromosomes. Several linkage groups, including LG1, LG4, LG11 and LG9, displayed relatively higher proportions of hypermethylated regions, indicating a cultivar-specific chromosomal distribution of methylation changes.

In the combined Khalas–Kheneizi analysis, the chromosomal distribution reflected contributions from both cultivars, with LG1, LG4, and LG8 consistently representing a higher proportion of total DMRs. Across all datasets, both hypermethylated and hypomethylated DMRs were detected on every chromosome, indicating that tissue culture-associated DNA methylation changes are distributed genome-wide rather than confined to specific chromosomal regions.

**Fig. 3:**
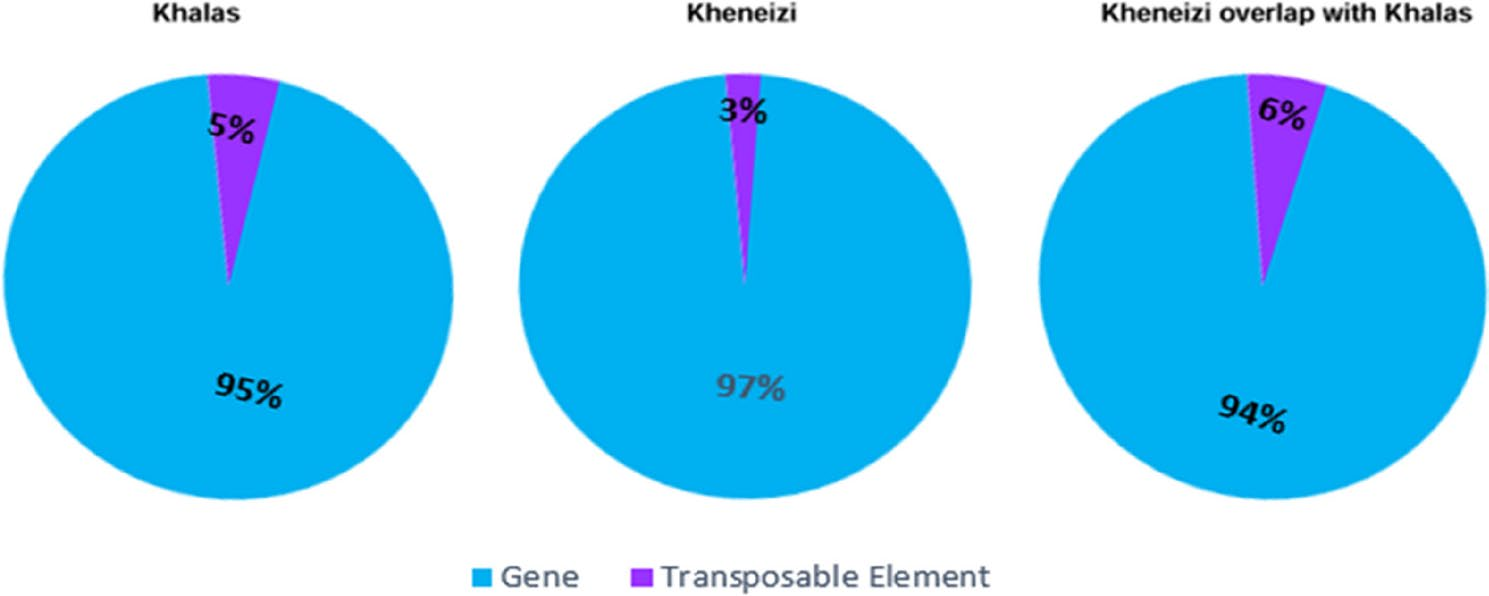
Distribution of differentially methylated regions (DMRs) across gene-associated and transposable element (TE) regions in date palm. Pie charts show the proportion of DMRs overlapping annotated gene-associated regions and transposable elements (TEs) in the Khalas cultivar (left), the Kheneizi cultivar (middle), and the combined Khalas–Kheneizi dataset (right). DMRs were classified based on their overlap with genomic features annotated in the *Phoenix dactylifera* PDK50 reference genome. Gene-associated regions include DMRs located within gene bodies or upstream regulatory regions, whereas TE-associated DMRs correspond to regions annotated as transposable element genes. Percentages represent the relative contribution of each category within each dataset

To further characterize the genomic distribution of differential DNA methylation, DMRs were classified according to their overlap with annotated gene bodies and transposable elements (TEs). Across both cultivars, the majority of DMRs overlapped gene-associated regions, whereas a smaller proportion mapped to transposable elements (Fig. 3). In the Khalas cultivar, approximately 95% of DMRs were located within gene-associated regions and 5% within TEs, while in the Kheneizi cultivar, gene-associated DMRs accounted for 97% and TE-associated DMRs for 3%. A similar distribution was observed in the combined dataset.

### Identification of the differentially methylated genes related to the DMR regions

**Fig. 4:**
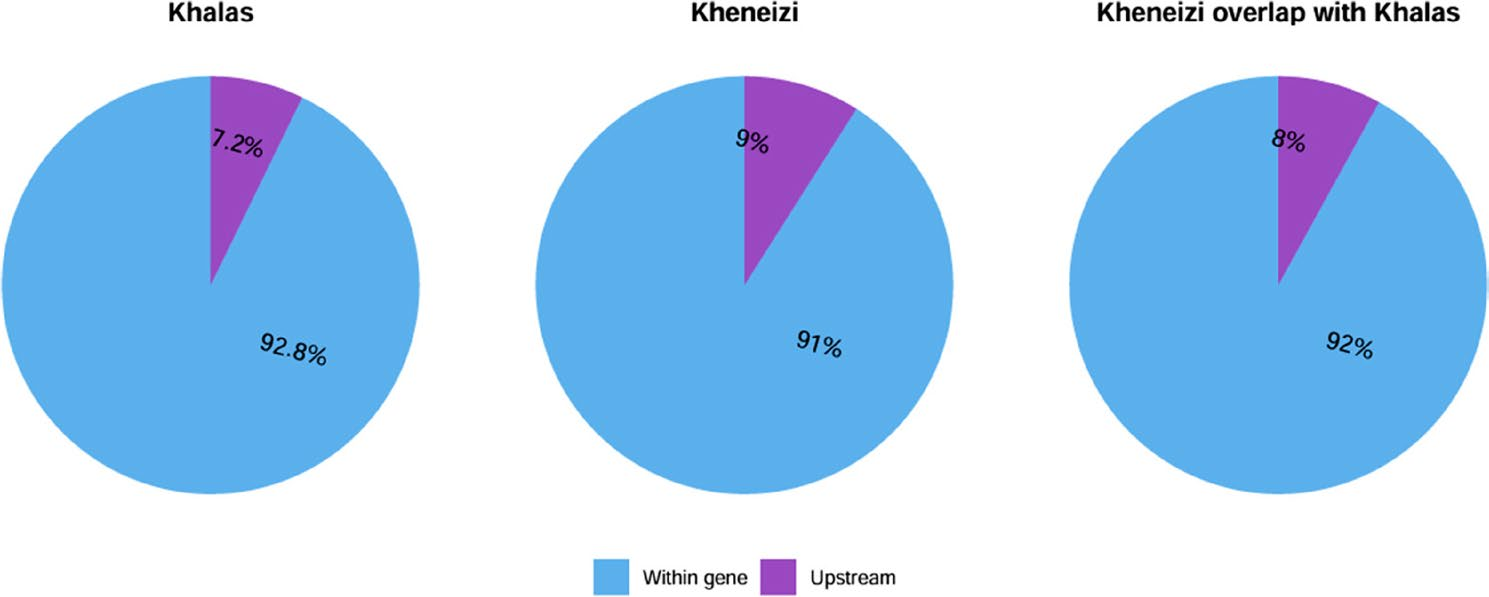
Distribution of differentially methylated region–associated genes (DMGs) within gene bodies and upstream regions in Khalas, Kheneizi, and the combined dataset. Pie charts show the proportion of DMGs located within gene bodies or in upstream regulatory regions in the Khalas cultivar, the Kheneizi cultivar, and the combined Khalas–Kheneizi dataset. DMGs were defined as genes whose gene body or upstream region overlapped with at least one differentially methylated region (DMR). Percentages indicate the relative distribution of DMGs across the two genomic categories within each dataset

Differentially methylated region–associated genes (DMGs) were defined as genes whose gene body or upstream regulatory regions overlapped with the identified DMRs. In the Khalas cultivar, the majority of DMGs were located within gene bodies (92.78%), whereas a smaller proportion (7.22%) overlapped upstream regions (Fig. 4). A similar distribution pattern was observed in the Kheneizi cultivar, where 91% of DMGs were located within gene bodies and 9% within upstream regions. Consistent with these cultivar-specific results, the combined analysis of Khalas and Kheneizi showed that 92% of DMGs were located within gene bodies and 8% within upstream regulatory regions.

### Functional analysis of methylated genes (DMGs)

The functional annotation provided an overview of the biological roles of the differentially methylated genes (DMGs). Enrichment of Gene Ontology (GO) terms was assessed using Fisher’s exact test (*p* < 0.05) to identify significantly enriched categories. The analysis was performed separately for hypermethylated and hypomethylated DMGs in both Khalas and Kheneizi cultivars. GO analysis classified the annotated sequences into three hierarchical ontologies, revealing their participation in Biological Process (BP), Molecular Function (MF), and Cellular Component (CC).

In the Khalas cultivar, hypermethylated DMGs in the BP category were significantly enriched (*p* < 0.05) in organelle organization, cellular component organization/biogenesis, and chromosome organization, with additional enrichment in macromolecule methylation, positive regulation of transcription by RNA polymerase II, and gene expression. These results indicate enrichment of genes involved in organelle organization and epigenetic regulation of transcription. In the CC category, the most enriched terms were organelle lumen, nuclear lumen, intracellular membraneless organelles, and protein-containing complexes, reflecting a predominant nuclear and subnuclear localization of the associated genes. For MF, the DMGs were mainly associated with ATP-dependent activity, catalytic activity on RNA, histone-modifying activity, and S-adenosylmethionine-dependent methyltransferase activity, indicating roles in chromatin dynamics and transcriptional regulation (Figure S1, Table S1). Collectively, this reflects the strong representation of genes involved in nuclear organization, chromatin remodelling, and epigenetic control of gene expression.

For hypomethylated DMGs in Khalas, BP enrichment was mainly observed in plant-type hypersensitive response, symbiont-induced defense-related programmed cell death, innate immune response, and response to singlet oxygen, indicating roles in defense and stress adaptation. Additional enriched terms included immune response, cell death, and immune system process. In the CC category, enriched terms included symplast, plasmodesma, cell junctions, and plasma membrane, reflecting the association of hypomethylated genes with intercellular communication and defense-related structures (Figure S2, Table S2). These results point to a strong involvement of hypomethylated genes in defense responses and cell-to-cell signaling. Overall, the enrichment analysis of DMGs in Khalas shows their participation in the regulation of developmental processes, epigenetic regulation of transcription, and stress- and defense-related pathways.

In the Kheneizi cultivar, hypermethylated DMGs in the BP category were significantly enriched in nucleic acid catabolic process, RNA catabolic process, regulation of protein metabolic process, RNA processing, translation, and post-transcriptional regulation of gene expression, indicating strong involvement in RNA metabolism and protein synthesis. In the CC category, enrichment was observed in cytoplasm, protein-containing complexes, and intracellular organelles, reflecting predominant cytoplasmic localization. For MF, enriched categories included translation factor activity, RNA binding, protein binding, metal ion transmembrane transporter activity, and various ATP-dependent enzymatic activities, pointing to roles in translation regulation, RNA interactions, and energy-dependent processes (Figure S3, Table S3).

For hypomethylated DMGs in Kheneizi, BP enrichment included broad terms such as cellular process, metabolic process, and cellular response to stimulus, together with more specific categories including phosphorylation, response to xenobiotic stimulus, and macromolecule modification, indicating roles in metabolism and stress responses. In the CC category, the most enriched terms were cytoplasm, intracellular organelles, membrane-bounded organelles, and protein-containing complexes. For MF, enrichment was observed in catalytic activity, kinase and phosphatase activity, ATP hydrolysis activity, and metal ion transmembrane transport, highlighting the roles of these genes in enzymatic regulation and phosphorylation-dependent processes (Figure S4, Table S4). These results indicate that hypomethylated DMGs in Kheneizi are mainly associated with metabolism, phosphorylation-mediated regulation, and energy-dependent enzymatic activity. Overall, the enrichment analysis of DMGs in Kheneizi indicates their involvement in RNA metabolism, translation, enzymatic activity, and cellular metabolic processes.

Taken together, the functional annotation revealed cultivar-specific enrichment patterns. In Khalas, DMGs were mainly associated with developmental processes, epigenetic regulation of transcription, and defense-related pathways, whereas in Kheneizi, they were enriched in RNA metabolism, translation, and cellular metabolic processes. Despite these differences, both cultivars showed common enrichment in protein-containing complexes and organelle-associated categories.

**Fig. 5:**
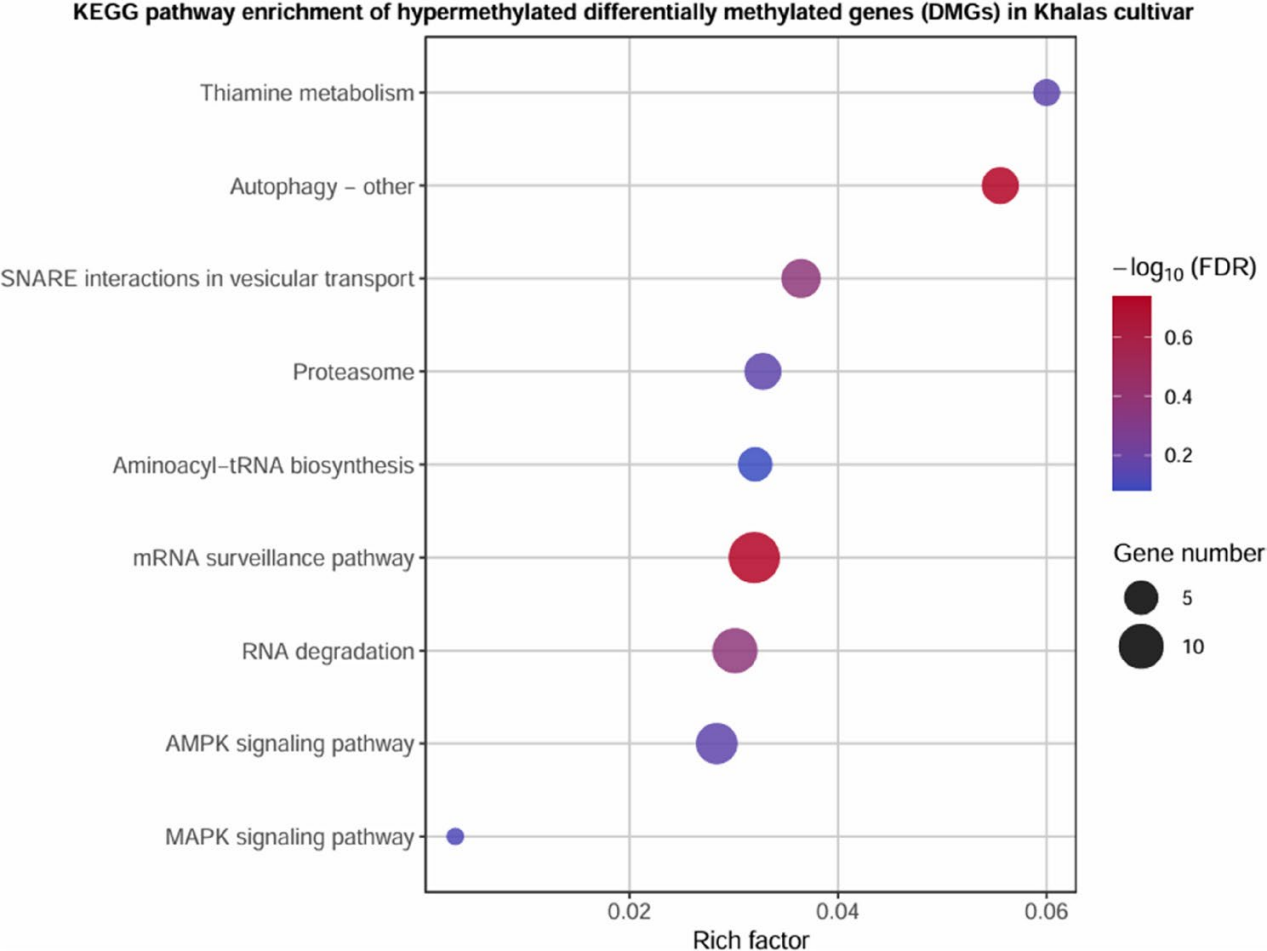
KEGG pathway enrichment analysis of hypermethylated differentially methylated genes (DMGs) in the Khalas cultivar. Bubble plot showing significantly enriched KEGG pathways among hypermethylated DMGs identified in tissue culture-derived plants relative to offshoot-derived controls. The x-axis represents the rich factor (ratio of DMGs associated with a given pathway to the total number of genes annotated in that pathway). Bubble size corresponds to the number of DMGs mapped to each pathway, and bubble color indicates the significance level expressed as − log₁₀(FDR). Only pathways passing the enrichment significance threshold are shown

KEGG enrichment analysis further revealed that in the Khalas cultivar, hypermethylated genes were significantly enriched (*p* < 0.05) in pathways related to protein synthesis (aminoacyl-tRNA biosynthesis, thiamine metabolism) and RNA-related processes (mRNA surveillance, RNA degradation, autophagy), highlighting potential impacts on gene expression regulation. Additional enrichment in MAPK signaling, AMPK signaling, the proteasome, and SNARE transport reflects roles in stress signaling, energy regulation, and protein turnover (Fig. 5).

**Fig. 6:**
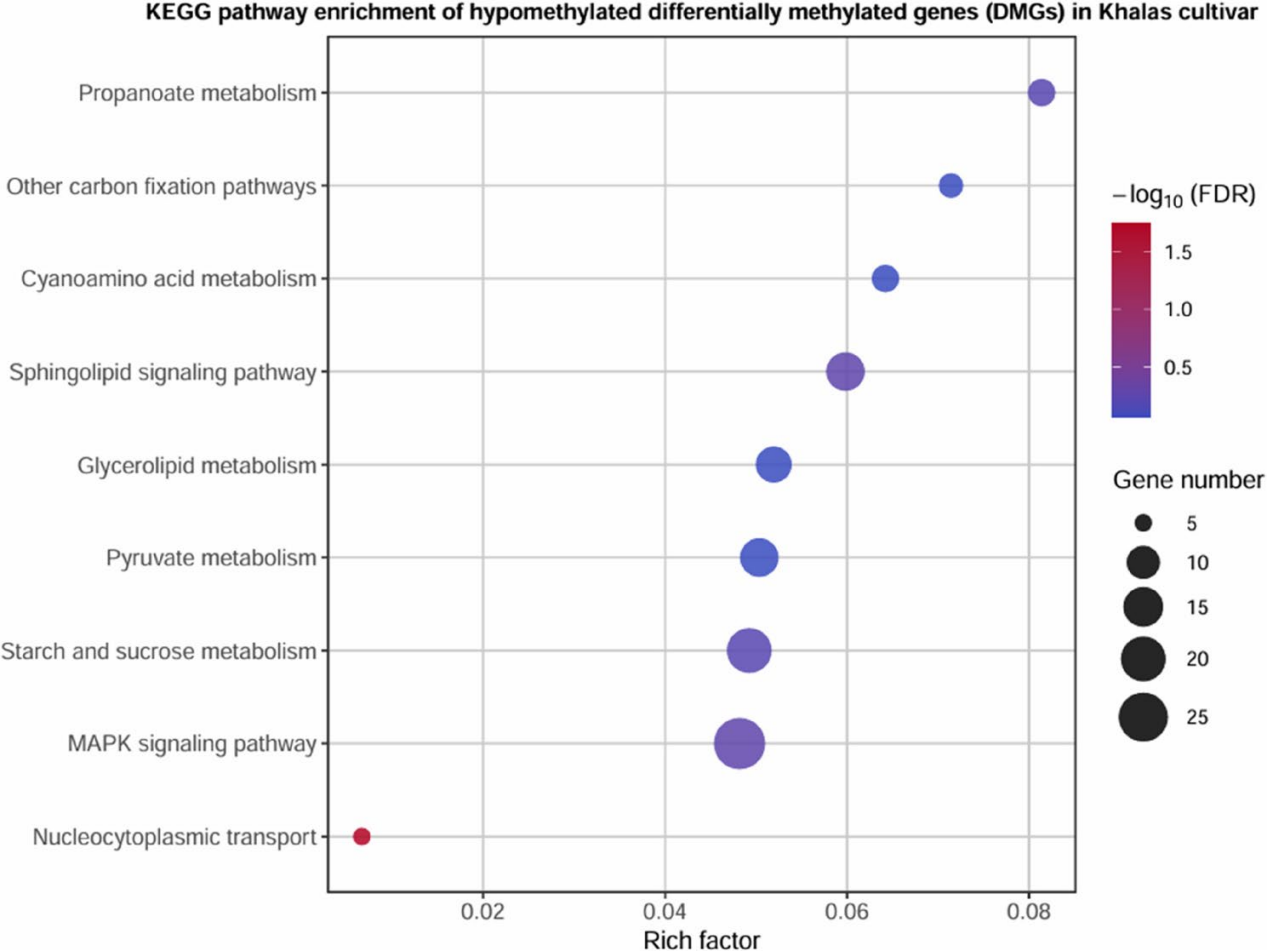
KEGG pathway enrichment analysis of hypomethylated differentially methylated genes (DMGs) in the Khalas cultivar. Bubble plot showing significantly enriched KEGG pathways among hypomethylated DMGs identified in tissue culture-derived plants relative to offshoot-derived controls. The x-axis represents the rich factor (ratio of hypomethylated DMGs associated with a given pathway to the total number of genes annotated in that pathway). Bubble size corresponds to the number of DMGs mapped to each pathway, and bubble color indicates the significance level expressed as − log₁₀(FDR). Only pathways meeting the enrichment significance threshold are displayed

Hypomethylated genes were enriched in metabolic and signaling pathways including starch and sucrose metabolism, carbon fixation, and cyanoamino acid metabolism, with additional enrichment in glycerolipid metabolism, pyruvate metabolism, sphingolipid signaling, MAPK signaling, and propanoate metabolism (Fig. 6).

Overall, DNA methylation changes in Khalas were associated with pathways related to metabolism, stress signaling, and RNA processing/gene expression quality control.

**Fig. 7:**
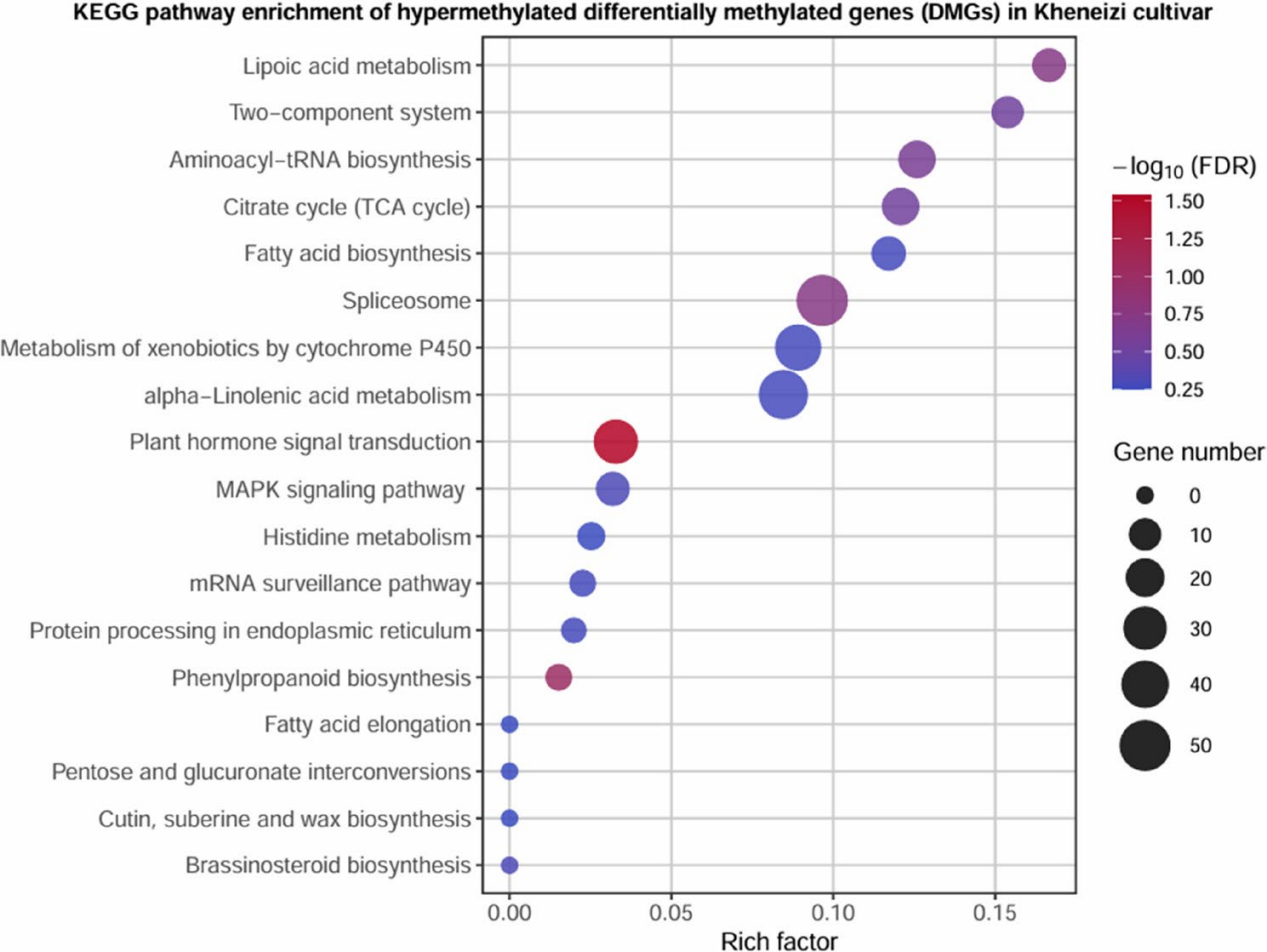
KEGG pathway enrichment analysis of hypermethylated differentially methylated genes (DMGs) in the Kheneizi cultivar. Bubble plot illustrating significantly enriched KEGG pathways among hypermethylated DMGs identified in tissue culture-derived plants relative to offshoot-derived controls. The x-axis represents the rich factor (ratio of hypermethylated DMGs associated with a given pathway to the total number of genes annotated in that pathway). Bubble size indicates the number of DMGs mapped to each pathway, while bubble color reflects the enrichment significance expressed as − log₁₀(FDR). Only pathways passing the enrichment significance threshold are shown

In the Kheneizi cultivar, hypermethylated genes were enriched in hormone- and stress-related pathways, notably plant hormone signal transduction, MAPK signaling, and brassinosteroid biosynthesis, alongside secondary metabolic pathways such as phenylpropanoid biosynthesis, cutin and wax biosynthesis, and α-linolenic acid metabolism. Broader regulatory processes including fatty acid metabolism, the spliceosome, and mRNA surveillance were also significantly enriched (Fig. 7).

**Fig. 8:**
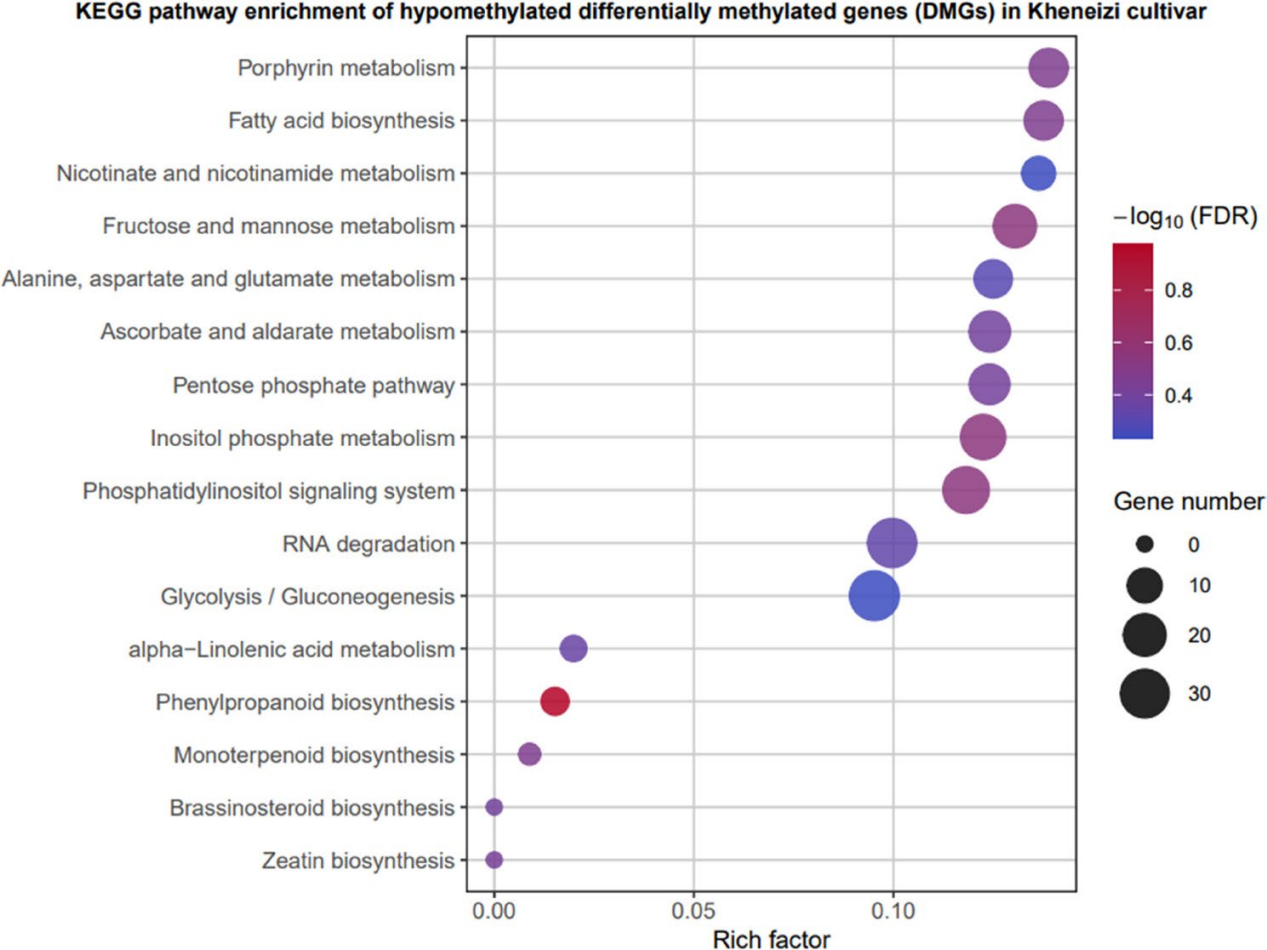
KEGG pathway enrichment analysis of hypomethylated differentially methylated genes (DMGs) in the Kheneizi cultivar. Bubble plot illustrating significantly enriched KEGG pathways among hypermethylated DMGs identified in tissue culture-derived plants relative to offshoot-derived controls. The x-axis represents the rich factor (ratio of hypermethylated DMGs associated with a given pathway to the total number of genes annotated in that pathway). Bubble size indicates the number of DMGs mapped to each pathway, while bubble color reflects the enrichment significance expressed as − log₁₀(FDR). Only pathways passing the enrichment significance threshold are shown

Hypomethylated genes were enriched in pathways linked to hormone biosynthesis (brassinosteroid and zeatin biosynthesis) and secondary metabolism (phenylpropanoid and monoterpenoid biosynthesis), together with redox- and energy-related processes such as α-linolenic acid metabolism, ascorbate metabolism, the pentose phosphate pathway, fructose/mannose metabolism, fatty acid biosynthesis, and porphyrin metabolism. Conserved pathways including glycolysis, RNA degradation, and inositol signaling were also enriched (Fig. 8).

Overall, DNA methylation changes in Kheneizi were associated with pathways related to hormone signaling, secondary metabolism, and gene expression regulation.

Across both cultivars, common enrichment was observed in mRNA surveillance, RNA degradation, MAPK signaling, and aminoacyl-tRNA biosynthesis, underscoring shared epigenetic regulation of stress signaling, protein synthesis, and RNA quality control in tissue culture-derived date palms. While Khalas showed stronger enrichment in primary metabolic processes, Kheneizi was more strongly enriched in hormone biosynthesis and secondary metabolism, reflecting cultivar-specific responses to DNA methylation changes.

## Discussion

Tissue culture propagation is widely recognized as an efficient technique for producing large numbers of true-to-type plants identical to their parental lines. However, the large-scale application of in vitro clonal propagation in date palm has also revealed the occurrence of off-type individuals that deviate from the original cultivar characteristics. Such abnormalities are generally attributed to somaclonal variation, which can arise from genetic mutations and/or epigenetic modifications acquired during the multiplication process. Previous studies have indicated that DNA methylation is strongly associated with somaclonal variation, as demonstrated in oil palm where altered methylation patterns were linked to the mantled phenotype [17, 18]. Similarly, DNA methylation induced during callus culture of pineapple may lead to somaclonal variation [30].

In this study, whole-genome bisulfite sequencing (WGBS) was employed to investigate and detect propagation-associated DNA methylation patterns by comparing phenotypically normal in vitro–derived plants and offshoot-derived plants of two widely cultivated date palm (*Phoenix dactylifera* L.) cultivars in Qatar, Khalas and Kheneizi.

Genome-wide DNA methylation levels showed modest but reproducible differences between tissue culture-derived and offshoot-derived plants across cytosine contexts. In both cultivars, CG methylation levels were slightly reduced in tissue culture-derived plants, whereas CHG methylation levels were higher. CHH methylation represented a minor fraction of total methylation and displayed cultivar-specific trends, being reduced in tissue culture-derived Khalas plants but higher in tissue culture-derived Kheneizi plants.

Despite the small magnitude of the observed differences, the consistent trends in global methylation across cytosine contexts suggest that in vitro propagation may influence genome-wide methylation patterns, even in phenotypically normal plants. At a finer genomic scale, these modest global changes were accompanied by the detection of thousands of DMRs in both cultivars.

Khalas exhibited a predominance of hypomethylated regions, whereas Kheneizi displayed slightly more hypermethylated regions (Table 2). The pattern observed in Khalas aligns with findings in rice regenerated plants, where the rate of hypomethylation was higher than that of hypermethylation [31]. However, in pineapple, consistent DMRs were associated with increased methylation levels, resembling the pattern observed in Kheneizi [30].

Across all datasets, CG sites tended to be hypomethylated, while CHG sites were more frequently hypermethylated. These findings suggest that tissue culture modifies cytosine methylation patterns in date palm, with a general reduction in CG methylation and a relative increase in CHG methylation in regenerated plants. Interestingly, the opposite trend was reported in pineapple, where callus-derived variants exhibited CG hypermethylation and CHG hypomethylation [30], suggesting that the direction of methylation change may be species or system dependent. By contrast, other studies have described more uniform methylation decreases across both CG and CHG contexts, such as in cassava [32] and maize [33]. Hence, methylation patterns vary across plant species.

In the CHH context, differential methylation was detected only in the Kheneizi cultivar, occurred at low frequency, and was predominantly hypomethylated, indicating limited and cultivar-specific variation in this methylation context.

The limited occurrence of CHH methylation changes observed in this study is consistent with the dynamic nature of CHH methylation previously reported in angiosperms [34]. In addition, the predominance of CHH hypomethylation aligns with findings reported in Moso bamboo, where reduced CHH methylation levels were associated with aging pathway genes involved in the regulation of numerous flowering-related genes [35].

The cultivar-specific presence of CHH DMRs in Kheneizi further suggests potential differences in epigenetic plasticity between genotypes, which may influence their response to in vitro propagation conditions.

Overall, the two cultivars exhibited distinct epigenetic responses to tissue culture propagation. While Khalas showed a predominance of hypomethylated regions, Kheneizi displayed a relatively higher proportion of hypermethylated regions, indicating different patterns of epigenetic reprogramming. These observations suggest that genotype plays a key role in shaping the direction and extent of methylation changes induced by tissue culture, reflecting cultivar-specific epigenetic plasticity.

The chromosomal distribution of DMRs revealed that tissue culture-induced methylation changes were not uniformly distributed across the genome but occurred across all chromosomes, with cultivar-specific biases in the relative proportions of hyper and hypomethylated regions.

Most DMRs were mainly associated with gene-related regions rather than transposable elements, indicating that tissue culture-induced methylation changes preferentially affect genic regions, essential for growth, development, metabolism, and stress responses structure with potential regulatory relevance [36]. Nevertheless, despite their lower representation, DMRs overlapping transposable elements should not be overlooked, as DNA methylation changes in TEs can spread into neighboring genic regions and influence gene expression. Indeed, methylation spreading around transposable elements has been shown to silence genes encoding zinc-finger BED domain proteins, such as RICESLEEPER2, with consequences for genome stability and developmental timing [37].

The functional annotation of DMGs, also revealed cultivar-specific enrichment patterns.

In the Khalas cultivar, enriched GO terms highlighted processes related to epigenetic regulation, chromatin organization, and transcriptional control. The associated genes were predominantly localized to nuclear and protein complex–associated structures and functioned through ATP-dependent chromatin remodeling, histone/DNA methylation, and transcription factor interactions, processes known to be tightly regulated by DNA methylation [38]. Additional enrichment in immune responses, defense mechanisms, and cell-to-cell communication is consistent with evidence that DNA methylation dynamically regulates plant immunity and pathogen responses [39, 40]. These results suggest that DNA methylation changes in Khalas contribute both to chromatin-based transcriptional regulation and to the activation of stress- and defense-related processes.

In the Kheneizi cultivar, enriched GO terms emphasized RNA metabolism and protein synthesis, along with broader categories including cellular and metabolic processes, phosphorylation, and xenobiotic response. The associated genes were localized mainly to cytoplasmic compartments and protein-containing complexes, reflecting roles in RNA turnover, translation, and energy-dependent enzymatic processes. Altogether, these results suggest that DNA methylation changes in Kheneizi primarily affect post-transcriptional regulation, translational control, and metabolic regulation.

Taken together, the GO analysis indicates that hypermethylated DMGs in both cultivars are primarily involved in gene expression regulation and metabolic processes, while hypomethylated subsets participate in defense-related pathways in Khalas and phosphorylation-mediated regulation in Kheneizi. These findings are consistent with previous reports showing that DNA methylation influences chromatin-based transcriptional control, RNA metabolism, and translational regulation in plants [41, 42].

The KEGG enrichment analysis revealed that in Khalas, hypermethylated DMGs were mainly linked to pathways involved in protein synthesis, RNA quality control, and stress signaling, whereas hypomethylated genes were enriched in primary metabolic processes. These patterns suggest that methylation changes in Khalas may contribute to altered growth and stress physiology through a combined impact on transcriptional regulation, metabolism, and defense responses.

In Kheneizi, hypermethylated DMGs were primarily associated with pathways involved in hormone signaling, stress responses, and secondary metabolism, while hypomethylated genes were enriched in hormone biosynthesis, redox regulation, and energy-related processes. These patterns suggest that methylation changes in Kheneizi may influence developmental regulation and stress adaptation through a combined impact on hormone signaling networks, secondary metabolic pathways, and transcriptional regulation.

Khalas and Kheneizi displayed clear differences in their KEGG enrichment patterns, suggesting cultivar-specific epigenetic responses. Despite these differences, both cultivars also converged on core functional categories, with enrichment in processes related to gene expression regulation, RNA regulation, protein synthesis, and stress signaling [43–45], ultimately suggesting that DNA methylation influences plant growth and stress responses. In date palm (*Phoenix dactylifera* L.), DNA methylation has been implicated in the plant’s response to salinity stress. Al-Harrasi et al. [46] demonstrated that salinity exposure led to differential methylation patterns in the roots of date palm, affecting gene expression profiles associated with stress responses. This suggests that DNA methylation contributes to the plant’s ability to adapt to saline environments by modulating the expression of stress-responsive genes. Similarly, significant alterations in both DNA methylation and gene expression were demonstrated in *Arabidopsis thaliana* plants in response to stress conditions [47]. Sikdar et al. [48] reported higher DNA methylation levels in tissue cultured lingonberry plants compared to cutting-propagated ones, accompanied by lower secondary metabolite accumulation, suggesting a potential inverse relationship between methylation and metabolite production.

Collectively, the GO and KEGG enrichment results point to DNA methylation as a regulator of developmental and stress-related processes in date palm. Given that many of these pathways are mediated by transcription factors (TFs), methylation-driven modulation of TF activity likely represents a pivotal mechanism underlying tissue culture-induced variation. For example, methylation at MADS-box gene promoters has been associated with delayed flowering in late-flowering ecotypes of *Arabidopsis* [49]. Similarly, DNA methylation can regulate auxin response factor (ARF) genes and components of auxin signaling pathways, thereby modulating auxin responsiveness during embryogenesis and organ formation [50].

In addition to developmental regulation, TF methylation has also been implicated in plant stress adaptation. For instance, members of major TF superfamilies were differentially methylated in wild strawberry under stress conditions, underscoring their role in coordinating stress-response networks [51]. Likewise, induction of DNA methylation in stress-responsive TFs has been documented in soybean under salinity stress [52], further highlighting the contribution of TF methylation to plant adaptive responses.

These TFs act at the top of regulatory hierarchies, and even subtle methylation changes at their loci can reshape downstream gene networks, ultimately influencing plant growth, reproduction, and responses to both biotic and abiotic stresses [53]. Therefore, the study of TF methylation is essential to understanding somaclonal variation in tissue culture-derived plants and may explain the emergence of off-types with developmental and reproductive abnormalities in date palm. In this context, Mattat et al. [54] reported that fruit set failure, commonly referred to as “Shess,” is among the most frequently encountered traits generated by epigenetic changes during the tissue culture process in Qatar. Alterations in DNA methylation of TFs governing the complex process of flowering may thus be directly related to pollination failure and the reduced fruit set rate observed in off-type plants and this possibility warrants further investigation.

Our findings demonstrate that tissue culture is associated with extensive and cultivar-specific DNA methylation alterations in date palm, revealing differential methylation patterns enriched in genes related to transcriptional regulation, metabolism, and stress-response pathways. These epigenetic changes may represent early molecular events associated with tissue culture-induced reprogramming and could potentially contribute to phenotypic variation observed such as fruit set failure (“Shess”) in regenerated plants. Importantly, the identification of differentially methylated transcription factors highlights potential master regulators through which methylation reprogramming can reshape developmental and reproductive processes. A deeper understanding of these mechanisms will not only expand fundamental knowledge of epigenetic regulation in perennial crops but also provide a foundation for developing strategies to minimize off-type formation and improve the stability and efficiency of clonal propagation systems in date palm.

However, the present study was not designed to establish a direct mechanistic link between DNA methylation changes and somaclonal variation. By focusing exclusively on phenotypically normal plants, our objective was to isolate propagation-associated epigenetic changes independent of visible abnormalities. While this approach enables the identification of early methylation reprogramming events, it does not allow direct association with phenotypic outcomes. Future studies incorporating off-type plants and analyses of within-group variability will be required to establish direct links between epigenetic modifications and phenotypic variation.

## Conclusion

This study demonstrates that tissue culture is associated with extensive and cultivar-specific DNA methylation alterations in date palm (*Phoenix dactylifera* L.), predominantly affecting CG and CHG contexts. The Khalas cultivar exhibited a predominance of hypomethylated regions, whereas Kheneizi showed a higher proportion of hypermethylated regions, with both cultivars displaying distinct distributions of differentially methylated genes across biological processes, molecular functions, and cellular components. Despite these cultivar-specific differences, shared enrichment of pathways related to RNA regulation, protein synthesis, and stress signaling suggests common epigenetic responses to in vitro propagation. Collectively, these results support that tissue culture-associated DNA methylation changes may represent an important epigenetic component potentially associated with somaclonal variation in date palm. A deeper understanding and characterization of DNA methylation dynamics in tissue culture systems may contribute to improved strategies for enhancing clonal fidelity, reducing undesirable variation, and optimizing large-scale propagation of date palm and other perennial crops.

## Supplementary Information

Supplementary Material 1.

## Data Availability

The whole-genome bisulfite sequencing (WGBS) data generated and analyzed during this study have been deposited in the NCBI Sequence Read Archive (SRA) under the BioProject accession number **PRJNA1353083** . The corresponding metadata and sequence files will be publicly available upon release. All other data supporting the findings of this study are available from the corresponding author upon reasonable request.

## Abbreviations

DNA: Deoxyribonucleic acid
TC: Tissue culture
WGBS: Whole genome bisulfite sequencing
DMR(s): Differentially methylated region(s)
DMG(s): Differentially methylated gene(s)
